# Spatial confinement effects of laser-induced breakdown spectroscopy at reduced air pressures

**DOI:** 10.1007/s12200-022-00020-9

**Published:** 2022-04-28

**Authors:** Zhongqi Hao, Zhiwei Deng, Li Liu, Jiulin Shi, Xingdao He

**Affiliations:** 1 School of Measuring and Optoelectronic Engineering, Nanchang Hangkong University, Nanchang, 330063 China; 2 Key Laboratory of Opto-electronic Information Science and Technology of Jiangxi Province, Nanchang Hangkong University, Nanchang, 330063 China; 3 Wuhan National Laboratory for Optoelectronics (WNLO), Huazhong University of Science and Technology, Wuhan, 430074 China

**Keywords:** Laser-induced breakdown spectroscopy (LIBS), Spatial confinement, Plasma temperature, Stark broadening

## Abstract

Spatial confinement is a simple and cost-effective method for enhancing signal intensity and improving the detection sensitivity of laser-induced breakdown spectroscopy (LIBS). However, the spatial confinement effects of LIBS under different pressures remains a question to be studied, because the pressure of the ambient gas has a significant influence on the temporal and spatial evolution of plasma. In this study, spatial confinement effects of LIBS under a series of reduced air pressures were investigated experimentally, and the plasma characteristics under different air pressures were studied. The results show that the reduced air pressure can lead to both earlier onset and weakening of the enhancement effect of the spatial confinement on the LIBS line intensity. When the air pressure drops to 0.1 kPa, the enhancement effect of the emission intensity no longer comes from the compression of the reflected shock wave on the plasma, but from the cavity’s restriction of the plasma expansion space. In conclusion, the enhancement effect of spatial confinement technology on the LIBS is still effective when the pressure is reduced, which further expands the research and application field of spatial confinement technology.

**Graphical abstract:**

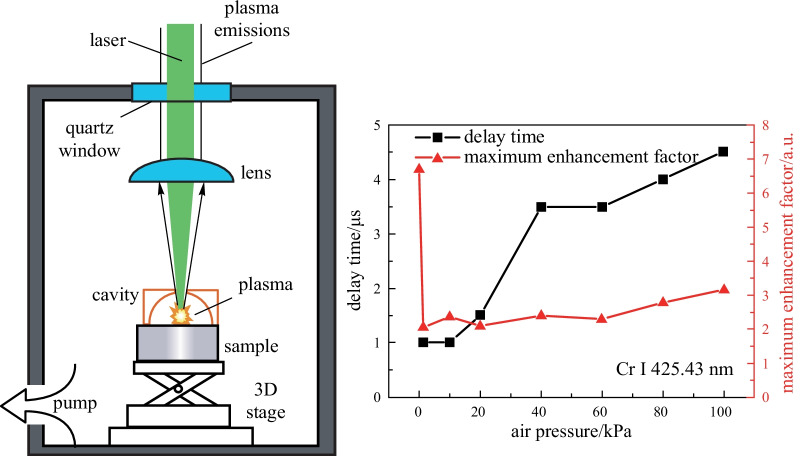

## Introduction

Laser-induced breakdown spectroscopy (LIBS) is a potential spectroscopy technology based on plasma spectral analysis from samples [1]. LIBS has been drawing attention in the last two decades due to its capability of rapid response, in situ elemental analysis with low invasiveness, simultaneous multi-element detection, no requirement for complicated sample preparation, remote detection, and in situ real-time analysis [2, 3]. However, weak spectral signal and low detection sensitivity are two drawbacks of LIBS [4, 5]. To enhance LIBS signal intensity and improve its sensitivity, researchers have used LIBS combined with a variety of new methods such as dual-pulse excitation [6, 7], spark discharge [8], micro-torches [9], nanoparticle enhancement [10–13], magnetic confinement [14–17], spatial confinement [18–21], and other methods [22–24].

Double-pulse LIBS (DP-LIBS) is an effective method to enhance signal intensity and improve analytical sensitivity relative to conventional LIBS [6, 7]. But DP-LIBS increases complexity and cost of the LIBS, since it uses two lasers. Spark discharge assisted LIBS (SD-LIBS) can give sixfold enhancement in the signal-to-background ratio for Al and Cu targets when compared to LIBS alone for the same laser conditions [8], and SD-LIBS has also been used to improve sensitivity in the determination of P concentration in fertilizers due to plasma reheating by the discharge [25]. A commercial butane micro torch was used to enhance plasma optical emissions in LIBS. Nanoparticle enhanced LIBS (NELIBS) was used to obtain 1–2 orders of magnitude in LIBS signals by depositing metallic nanoparticles on metal samples [11, 26]. The basic mechanisms of NELIBS were reduction of the laser induced plasma excitation threshold of the sample using nanoparticles. Magnetic confinement is still the research hotspot of LIBS technology [17, 27], but the magnetic confinement effect is limited by insufficient magnetic field strength of available magnets. Compared with the above methods, spatial confinement is a simple and cost-effective method for enhancing signal intensity and improving the detection sensitivity of LIBS. In the past ten years, different cavity configurations have been studied for their confinement effects and quantitative analysis ability [18, 20, 21, 28–31]. However, all these experiments have been carried out in a normal air environment. Studies in other gaseous environments and under different pressures have not been reported.

In this study, the main aim was to investigate the spatial confinement effects for LIBS under reduced air pressures from 0.1 to 100 kPa. The enhancement effects of LIBS were first investigated by analyzing spectral lines of Cr in steel samples. To understand the mechanism of the spatial confinement effects, the temporal evolutions of the signal intensities at different pressures were studied, and the plasma temperature and Stark broadening of Fe I spectral lines (proportional to electron number density) under different experimental conditions were discussed in this paper.

## Experimental methods

**Fig. 1:**
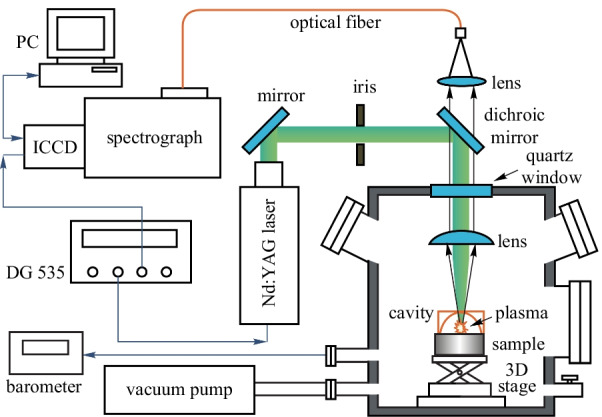
Schematic diagram of the experimental setup

The experimental setup used in the present studies is schematically shown in Fig. 1. The plasma was generated by a Q switched Nd:YAG laser (Beamtech, Nimma-400, 8 ns pulse duration) operating at 532 nm with pulse energy of 80 mJ and repetition rate of 3 Hz. The laser beam was reflected by a dichroic mirror (reflection band: about 474–554 nm), through the quartz window of the chamber and focused by a plano-convex lens (*f* = 150 mm); it then passed the 2-mm hole at the top of the hemispherical cavity (aluminum, diameter 5 mm) onto the steel target, with a focal point 4 mm below the target surface. The sample was mounted onto a motorized XYZ translation stage, and was moved to provide a fresh surface for each laser shot. The sample and the translation stage were put in a chamber where the air pressure could be tuned by a vacuum pump (KYKY Technology Co., LTD., RVP-6). A steel sample (GSB03-2582-2010-5#, with 0.171 wt% Cr) was used in this work. The pressure was measured using a barometer (Department of Electronics, Peking University, DL-4) linked with the chamber. The plasma emission was collected using an optical fiber through another UV-grade quartz lens placed on top of a dichroic mirror and coupled into a spectrometer. A Czerny-Turner spectrograph (Andor Technology, Shamrock 500i, three grating: 2400, 1800, and 1200 lines per mm, slit width: 10 μm to 2.5 mm) equipped with an intensified charge coupled device (ICCD) (Andor Technology, DH320T, sensor array size 1024 × 255, pixel size 26 μm) was used. The slit width of the spectrograph was set to 10 μm, and the 1200 lines/mm grating with the resolution of 0.08 nm at 435 nm was used in this study, its effective wavelength range was 200–865 nm. A digital delay generator (Stanford Research System, DG535, 5 ps delay resolution) was adopted to trigger the laser and control the gate delays of the ICCD. To reduce the influence of laser energy fluctuation on spectral intensities, each spectrum requires accumulation of plasma emissions from 20 laser shots. Every measurement was repeated six times, and the average data were used for analyses.

## Results and discussion

### Spatial confinement effects of LIBS at different air pressures

In general, along with the generation and expansion of the laser-induced plasma in air, a shock wave is generated. During the shock wave expansion, it is reflected when it encounters obstacles such as a plate wall or a cylindrical wall and compresses the plasma plume. The compressed plasma leads to an increased collision rate among the particles, resulting in an increased number of atoms in high-energy states and, hence, reheating and maintaining a higher temperature in the plasma to enhance the emission intensity.

**Fig. 2:**
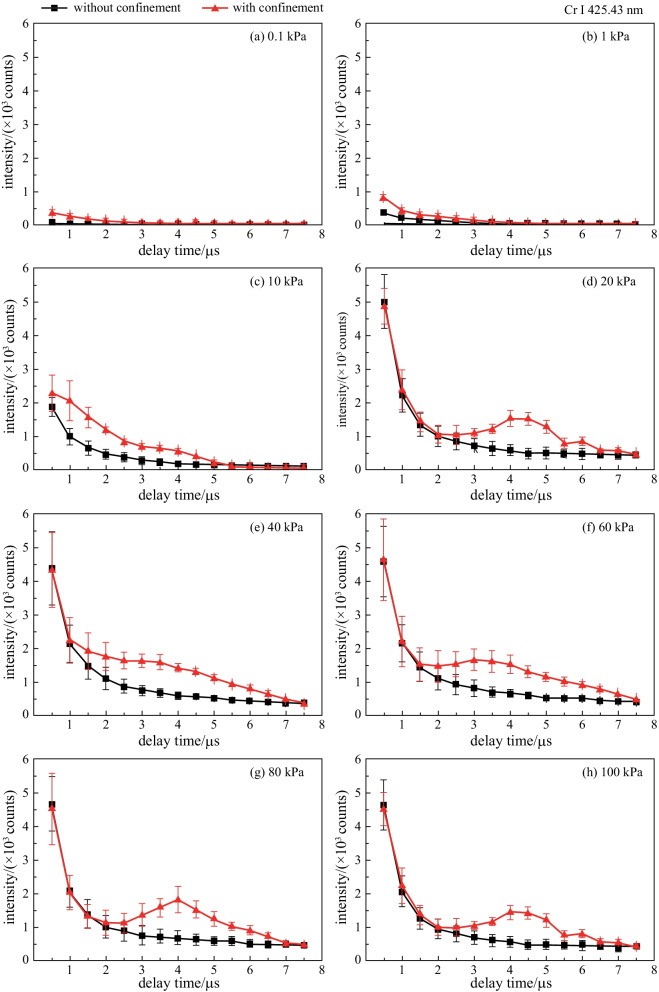
Spatial confinement effects at different air pressures. **a** 0.1 kPa, **b** 1 kPa, **c** 10 kPa, **d** 20 kPa, **e** 40 kPa, **f** 60 kPa, **g** 80 kPa, **h** 100 kPa

Figure 2 shows the temporal variation that was observed in emission intensity of Cr I 425.43 nm at various air pressure in the absence and presence of spatial confinement. The error indicated by the error bars may have originated from the pulse-to-pulse energy fluctuation and the deviation of the cavity position among different measurements. The intensity enhancement varied with acquisition delay time, and the time for the strongest enhancement effect appear gradually increased.

As shown in Fig. 2a, for the air pressure of 0.1 kPa, the spectral intensity of plasma emission spectrum significantly increased when the delay time was less than 3.5 μs with the action of the hemispherical cavity. In such a low-pressure environment, the spectral enhancement did not come from the compression of the reflected shock wave by the cavity, but was due to the restriction of the confinement cavity on the expansion space of the plasma plume. In addition, according to our previous research [32] and other literatures [33], if there is compression of the reflected shock wave on the plasma, the curve of the observed spectral line intensity with the acquisition delay will rise or fall slowly at a certain moment. However, the above phenomenon was not observed, as is shown in Fig. 2a. In addition, according to our previous research, when the air pressure drops to 1 kPa with pulse energy of 80 mJ at delay time of 0.5 μs, the radius of the plasma should already be larger than 2.5 mm, which is the radius of the cavity. As the air pressure increases, the confinement effect of the reflected shock wave by the cavity should become obvious gradually. After the air pressure was higher than 20 kPa, as shown in Fig. 2b–h, some enhancement peaks appeared with confinement at about 2.5, 3, 3.5, 4, and 4.5 μs for air pressure of 20, 40, 60, 80, and 100 kPa.

**Fig. 3:**
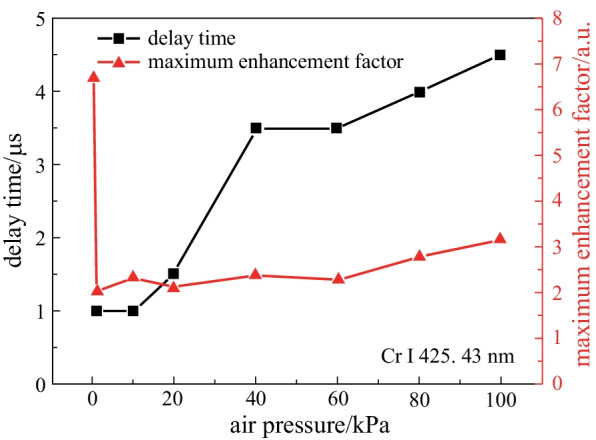
Maximum enhancement factors and its corresponding delay times at different air pressures

The maximum enhancement factors and corresponding delay times at different air pressures are given in Fig. 3. The maximum enhancement factor was 6.7 at delay time of 1 μs for air pressure of 0.1 kPa due to the confinement effect of the cavity on the expanding plasma. As the air pressure increased, the confinement effect of the air on the plasma plume increased, and the expansion speed of the plasma plume decreased. The confinement cavity did not directly affect the plasma plume, but confined the plasma by reflecting the shock wave. For this reason, with the air pressure increasing from 1 to 100 kPa, the maximum enhancement factors increased from 2.05 to 3.15, and their corresponding delay time gradually increased from 1 to 4.5 μs. The results show that the increase of atmospheric pressure caused the time lag of the confinement enhancement effect, and the increase of air density made the reflected shock wave have enough energy to compress the plasma.

To understand the dynamics of the plasma, a drag model and a shockwave model were used. For low background pressures, the classical drag force model shows better agreement with the dynamics of the plasma than the shockwave model. The expansion radius of the plume is described by a drag model given by [34]

1 $$R = R_{0} \left[ {1 - \exp \left( { - \beta t} \right)} \right],$$

where *R*_0_ is the maximum expansion radius of plasma plume, *β* is a slowing coefficient ($R_{0} \beta = v_{0}$), and *v*_0_ is the initial expansion velocity.

For high background pressures, a shockwave model is used usually. The propagation of the shock front by the background gas follows the Taylor-Sedov theory, which describes the distance-time relation of shockwave propagation from a point explosion [34]:

2 $$R = \xi_{0} \left( {{{E_{0} } \mathord{\left/ {\vphantom {{E_{0} } {\rho_{0} }}} \right. \kern-\nulldelimiterspace} {\rho_{0} }}} \right)^{{{1 \mathord{\left/ {\vphantom {1 5}} \right. \kern-\nulldelimiterspace} 5}}} t^{{{2 \mathord{\left/ {\vphantom {2 5}} \right. \kern-\nulldelimiterspace} 5}}} ,$$

where *R* is the expansion distance of shockwave from the target surface, $\xi_{0}$ is a constant depending on the specific heat capacity ratio, *E*_0_ is the amount of energy released during the explosion through a background gas of density $\rho_{0}$, and *t* is the delay time from the initiation of laser illumination. At the early stage of the expansion, both models are well adapted due to the shock wave and the plasma expands with an approximately the same velocity. The plume expansion at moderate pressure is represented by the shock model, and the drag model describes the plume expansion at reduced pressure. According to Eq. (2), the expansion distance *R* of the plume increased with decrease of the air pressure for the same condition of the laser energy and observation delay time.

The density of air varies with its pressure, resulting in different transmission speeds of the plume and shock waves. When the air pressure drops, the gas density decreases, the confinement effect of the air on the plasma is weakened, so the expansion speed of the plasma plume becomes faster. According to Eq. (1), the slowing coefficient *β* decrease with the increasing expansion speed of the plume, and the maximum expansion radius *R* of plasma plume decreases. Therefore, the reflection time of the shock wave by the cavity is shortened under a low air pressure, and the time for the appearance of the confinement enhancing effect is also shortened correspondingly. Both the expansion of plasma and the transmission of shock waves are affected by gas pressure and complex changes are produced during this process.

### Characteristics of spatial confinement plasma under different air pressures

To further study the physical mechanism of and spatial confinement plasma under different air pressures, the plasma temperature (*T*) and electron number density (*n*_e_) were investigated [35]. In this work, the temperatures *T* were estimated by the Boltzmann-plot of 13 Fe lines in the range of 418–442 nm. The atomic transition parameters of 13 Fe lines are listed in Table 1, where *λ* is the wavelength, *A*_*ij*_ is the radiative transition probabilities, *g*_*i*_ is the degeneracy of upper lever, *E*_*i*_, and *E*_*j*_ are the energies of upper and lower levers of the line, respectively.

**Table 1:** Atomic transition parameters of 13 Fe I lines

*λ*/nm	*A*_*ij*_*/*(× 10^7^ s^−1^)	*E*_*i*_/eV	*E*_*j*_/eV	*g*_*i*_
418.17	2.32	5.795	2.831	7
418.78	1.52	5.385	2.425	7
419.91	4.92	5.998	3.046	11
421.93	2.88	6.511	3.573	13
426.04	3.99	5.308	2.399	11
427.17	2.28	4.386	1.484	11
428.24	1.21	5.070	2.175	5
429.92	1.29	5.308	2.425	11
430.79	3.38	4.434	1.557	9
431.51	0.776	5.070	2.197	5
432.58	5.16	4.474	1.608	7
436.98	0.609	5.883	3.046	9
441.51	1.19	4.415	1.607	7

**Fig. 4:**
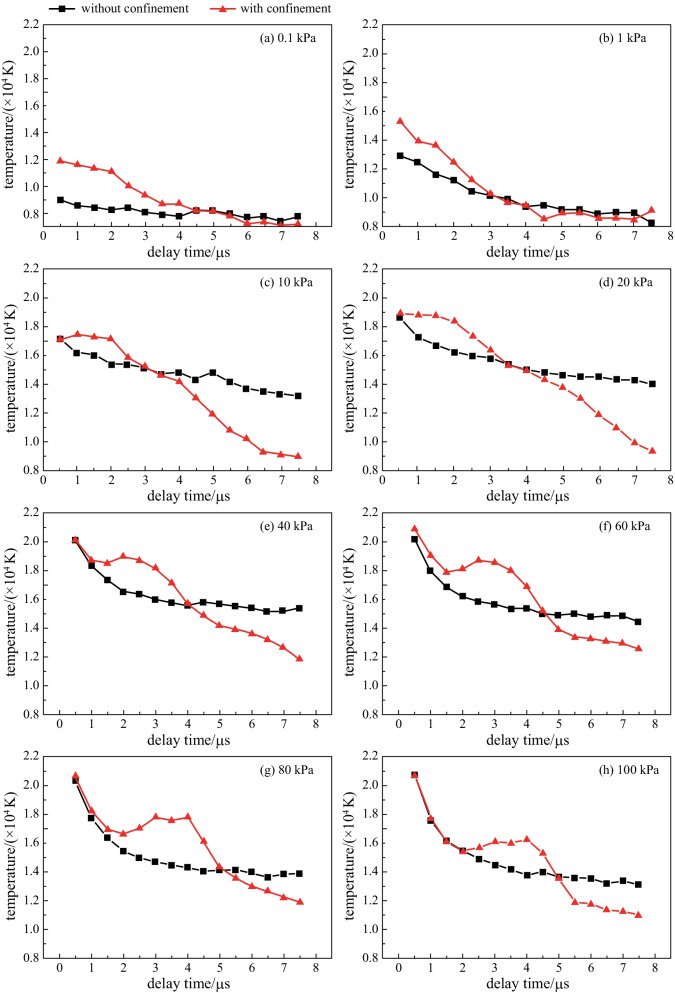
Temporal evolutions of plasma temperature under different air pressures. **a** 0.1 kPa, **b** 1 kPa, **c** 10 kPa, **d** 20 kPa, **e** 40 kPa, **f** 60 kPa, **g** 80 kPa, **h** 100 kPa

The temporal evolutions of plasma temperature under different air pressures are given in Fig. 4. The spatial confinement can effectively increase the temperature of the plasma, and be comparison with Fig. 2, it can be seen that the temporal evolutions of temperature were similar to those of the spectral intensity. Thus, the increase in plasma temperature is one of the reasons for the LIBS spectral enhancement. Spatial confinement effects came from the compression effect of the reflected shock waves on the expanded plasma, when the reflected shock wave was in contact with the plume and pushes the plume backward, the kinetic energy of the shock wave was converted to thermal energy of the plasma through the compression and collision of ambient gas [36]. Thus the spatial confinement could cause increase of plasma temperature. However, the spatial confinement cavity could not generate new energy. Therefore, as shown in Fig. 4, the heating time caused by the compression of the plasma plume by the spatial confinement was limited. In later delay times, the plasma temperature with the cavity fell more quickly and was even lower than that without cavity. This could possibly be explained as in the cavity case, the shockwave reflection and compression of the plasma being led to more violent convection of particles in plasma. The loss of energy caused its temperature to drop rapidly for later delay times. Similar results have also been discussed by Fu et al. [33].

The electron number density, *n*_e_, is proportional to the Stark broadening of the spectral line, so the value of *n*_e_ can be estimated accordingly[35]. Unfortunately, in the limited spectral range (417–444 nm) collected by the Czerny-Turner spectrograph, there are no lines suitable for *n*_e_ calculation due to the unavailability of a Stark broadening parameter of these lines in the existing literature. Thus the Stark broadening of Fe I 426.05 nm was used to estimate *n*_e_ in this work, because the Fe line is strong and undisturbed by other lines.

**Fig. 5:**
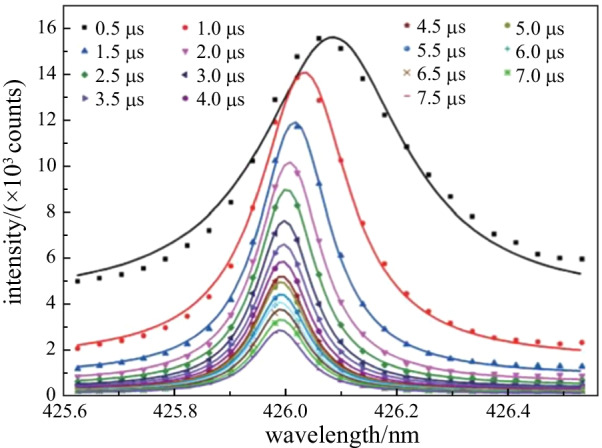
Lorentzian fitting of line Fe I 426.05 nm at 100 kPa for different delay times

Figure 5 shows the Lorentzian fitting of line Fe I 426.05 nm at 100 kPa for different delay times. The full width half-maximum (FWHM) and peak of the fitted profiles show obvious changes due to Stark broadening and shift, which was caused by the interaction of the emitting atoms with fast moving electrons and the slow-moving ions in the plasma. As can be seen in Fig. 5, the Stark shifts of the line gradually decreased with the increasing delay times. This was because the stark shifts of the spectral line were proportional to the electron number density, based on the following equation [37]:

3 $$\delta \lambda = d_{{\text{s}}} n_{{\text{e}}} \times 10^{ - 16} ,$$

where *d*_s_ is Stark shift parameter, and *n*_e_ (cm^−3^) is the number density of electrons in the plasma. The *n*_e_ decreased with the increasing delay times, and the Stark shifts was also reduced accordingly.

The slit width used in this work had a fixed value of 10 μm, so the instrumental width of the spectrograph at 426.05 nm was 0.08 nm, which could be found on the spectrograph manufacturer’s website. There may have been some errors in the values of instrument broadening, but it was a constant and did not affect the trend of Stark broadening. The values of the Stark broadening ($\Delta \lambda$) of Fe I 426.05 nm could be obtained by subtracting instrumental width from its FWHM.

**Fig. 6:**
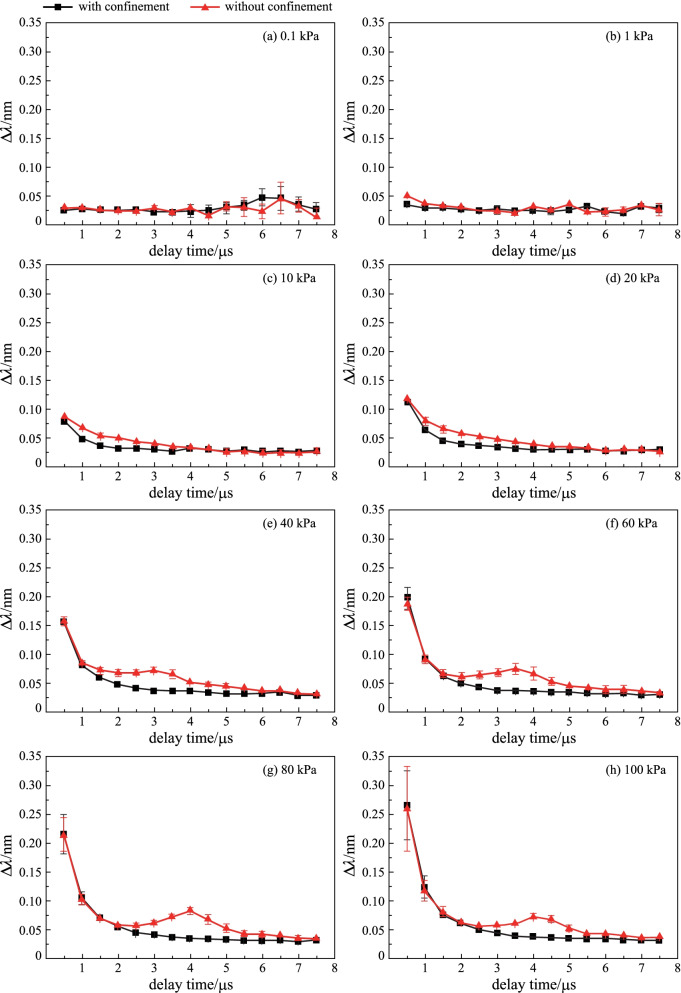
Temporal evolutions of Stark broadening ($\Delta \lambda$) of Fe I 426.05 nm at different air pressure. **a** 0.1 kPa, **b** 1 kPa, **c** 10 kPa, **d** 20 kPa, **e** 40 kPa, **f** 60 kPa, **g** 80 kPa, **h** 100 kPa

The $\Delta \lambda$ verses delay times at different air pressure with and without confinement are shown in Fig. 6. On the whole, with the delay time increasing, the values of $\Delta \lambda$ decreased, and the *n*_e_ also decreased. As can be seen in Fig. 6a, there was no obvious change in $\Delta \lambda$ when the air pressure was reduced to 0.1 kPa. The results once again showed that spatial confinement could not produce compression of the particles in the plasma plume at such a low pressure. When the pressure rose, the high-density air restricted the expansion of the particles in the plasma, and the shock wave had a high energy, which was enough to reflect back on the cavity wall to effectively compress the plasma plume, thereby causing the electron number density to increase.

In the later stages of the existence of plasma, the large consumption of plasma energy leads to a slow expansion speed and a slow decline in the electron number density. In addition, Fe I 426.05 nm is a strong line and has a long lifetime. Therefore, as shown in Fig. 6, the $\Delta \lambda$ decayed very slowly in the later stage of the plasma.

## Conclusions

The spatial confinement effects in LIBS under reduced air pressures from 0.1 to 100 kPa were investigated in detail. The temporal variations in emission intensity of Cr I 425.43 nm at various air pressures in the absence and presence of spatial confinement were experimentally studied. The research results show that, the enhancement effect of the emission intensity does not originate from the compression of the reflected shock wave on the plasma when the air pressure drops to 0.1 kPa, but from the direct limiting of the expansion space of the plasma plume by the cavity. When the gas pressure rose to 1 kPa, the confinement effect of the reflected shock wave on the plasma plume appeared. As the air pressure increased from 1 to 100 kPa, the maximum enhancement factor of the line intensity increased from 2.05 to 3.15, and their corresponding delay times gradually increased from 1 to 4.5 μs. The limited expansion space and the compression from the reflected shock wave made the plasma temperature and electron number density increase within a certain time delay range. Therefore, this work presents the influence of air pressure on the spatial confinement effect, and studies its physical mechanism from the plasma characteristics, which can provide a reference for further research and application of plasma confinement technology under different pressure environments.
